# Construction and Validation of Predictive Model to Identify Critical Genes Associated with Advanced Kidney Disease

**DOI:** 10.1155/2020/7524057

**Published:** 2020-11-12

**Authors:** Guangda Xin, Guangyu Zhou, Wenlong Zhang, Xiaofei Zhang

**Affiliations:** ^1^Department of Nephrology, China-Japan Union Hospital of Jilin University, Changchun, China; ^2^Department of Matological and Oncological, China-Japan Union Hospital of Jilin University, Changchun, China; ^3^Department of Pediatrics, China-Japan Union Hospital of Jilin University, Changchun, China

## Abstract

**Background:**

Chronic kidney disease (CKD) is characterized by progressive renal function loss, which may finally lead to end-stage renal disease (ESRD). The study is aimed at identifying crucial genes related to CKD progressive and constructing a disease prediction model to investigate risk factors.

**Methods:**

GSE97709 and GSE37171 datasets were downloaded from the GEO database including peripheral blood samples from subjects with CKD, ESRD, and healthy controls. Differential expressed genes (DEGs) were identified and functional enrichment analysis. Machine learning algorithm-based prediction model was constructed to identify crucial functional feature genes related to ESRD.

**Results:**

A total of 76 DEGs were screened from CDK vs. normal samples while 10,114 DEGs were identified from ESRD vs. CDK samples. For numerous genes related to ESRD, several GO biological terms and 141 signaling pathways were identified including markedly upregulated olfactory transduction and downregulated platelet activation pathway. The DEGs were clustering in three modules according to WGCNA access, namely, ME1, ME2, and ME3. By construction of the XGBoost model and dataset validation, we screened cohorts of genes associated with progressive CKD, such as *FZD10*, *FOXD4*, and *FAM215A*. *FZD10* represented the highest score (*F* score = 21) in predictive model.

**Conclusion:**

Our results demonstrated that *FZD10*, *FOXD4*, *PPP3R1*, and *UCP2* might be critical genes in CKD progression.

## 1. Background

Chronic kidney disease (CKD) is a syndrome characterized by persistent kidney function loss. Patients suffered from gradually reduced glomerular filtration rate (GFR) or kidney damage over many years and finally lead to end-stage renal disease (ESRD) [1]. An estimate of 10%-13% population is affected by CKD in the United States and in China, and the prevalence increases dramatically as the age prolonged [2]. Despite great improvements in understanding this disease, the exact molecular mechanism of CKD progression remains largely uncovered.

Genomic transcript analysis has been widely used in precision medicine studies, and it can provide an unbiased description of genome changes in disease progression. Previous studies have identified differentially expressed genes in ESRD. For example, diabetic nephropathy (DN) was a major cause of ESRD, and a recent study identified the candidate genes in the progression of DN by microarray dataset analysis [3]; cohorts of hub genes were screened related to disease progression, such as *COL6A3*, *MS4A6A*, and *PLCE1*. Dai et al. performed the gene coexpression network analysis and identified a series of hub genes associated with immune functions in ESRD patients [4]. These types of genomic profiles analysis could result in a highly robust prediction for CKD progression.

WGCNA facilitated the summary and normalization of methods and functions. Kim et al. successfully used the WGCNA method to identify differentially expressed miRNA in CKD to predict potential targets in CKD-mineral bone disorder [5]. In this study, we are aimed at exploring the genomic changes in CKD development and predict crucial factors under ESRD conditions by constructing a disease prediction model.

## 2. Materials and Methods

### 2.1. Data Resource

Microarray datasets under access number GSE97709 [6] and GSE37171 [7] were downloaded from the GEO database, which were associated with peripheral blood samples from subjects with CKD, ESRD, and healthy controls. GSE97709 included 28 ESRD samples, 8 CKD samples, and 12 normal samples. GSE37171 consisted of 75 ESRD samples and 40 normal samples. The gene profile (Release 26, grch38.p10) related to annotation information was also downloaded from the GENCODE database.

### 2.2. Screening the DEGs

Firstly, the microarray dataset GSE97709 were normalized using betaqn methods in R software. After data preprocessing, the limma package [8] was used to screen DEGs between CDK groups and normal groups. Thus, the major DEGs were also identified from ESRD samples compared with CDK samples. In order to reduce the false positives in sequencing results, Benjamini and Hochberg method was used to correct the *p* values. Adjusted *p* value ≤ 0.05 and ∣logFC | ≥1 were considered as thresholds.

### 2.3. Functional Enrichment Analysis for DEGs

Gene ontology (GO) biological terms (Biology Process; Molecular Function; and Cellular Component) were annotated using online tool DAVID [9] (version 6.7, https://david-d.ncifcrf.gov/), and the results were visualized using GOplot [10], an R package for visually combining expressed dataset with functional analysis. KEGG enrichment analysis was performed based on Gene set enrichment analysis (GSEA, version 3.0) [11, 12]. The results with adj.*p*.value ≤ 0.05 were considered as a significant difference.

### 2.4. Weighted Coexpression Network Analysis

In this study, we used the WGCNA package [13] (Version 1.61) to analyze the functional modules, construct the coexpression networks, identify gene cohorts, and calculate topological characteristics. The expressed association was first calculated to identify the correlation of two genes and the following adjacency function definition and module division. The modules contained more than 30 RNA and cutHeight = 0.99 was set as thresholds.

The expression data of DEGs were extracted from gene expression profiles together with the clinical characteristics of patients. Then, the data matrix was normalized using the betaqn method. Firstly, the input matrix was preprocessed by screening the top 75% of genes with higher median deviation values. The MAD values should be more than 0.01, and genes with deleted expression values were also removed. As for the network construction, the parameters were set as follows: gene numbers in modules should be more than thirty while correlation type (corType) was set as the Pearson Correlation Coefficient.

### 2.5. Construction of Disease Prediction Model

In order to explore the better subset of features for crucial genes prediction, we selected a new method for mining feature subset, such as XGBoost [14], Random Forest methods in the Sklearn library (https://scikit-learn.org/), and supervised classified method SVM. Firstly, we integrated two data sets GSE97709 and GSE37171 and extracted the coexpressed profile data as feature subsets, which contained 11,629 genes. GSE37171 dataset were mainly used for training and verification of classification models. After feature selection, we constructed and selected the optimal classified models by using the GridSearchCV method. Thus, the samples derived from the dataset could be predicted whether to be kidney disease according to their gene expression values. Here, we used the preprocessing.scale method for data normalization. Then, the samples were randomly divided into training set and testing set at a ratio of 6 : 4 (random_state = 123). Three-fold cross-validation was used to validate the performance of the training model and parameters of class_weight = “balanced” were added to eliminate the effect of classification imbalance. Finally, accuracy analysis and AUC area were considered as criteria for model evaluation on the test set (mainly AUC area).

## 3. Results

### 3.1. DEGs Screening and Cluster Analysis

**Figure 1:**
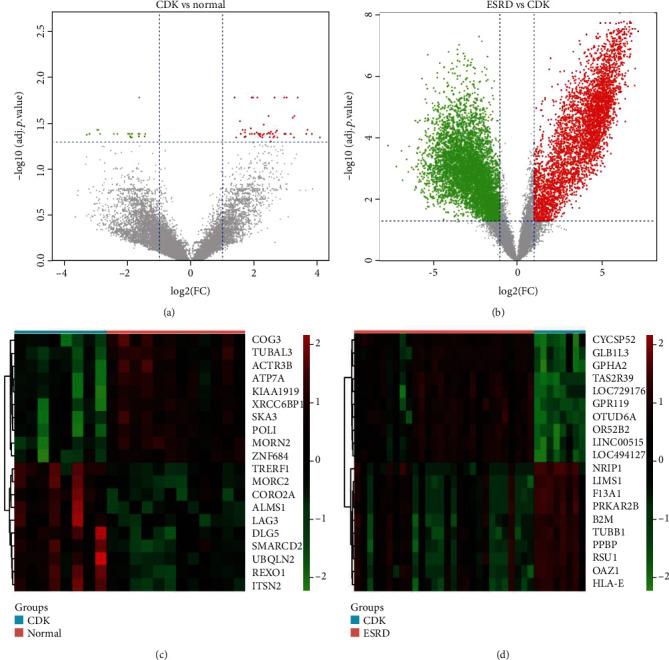
The volcano map and clustering analysis for the identification of differentially expressed genes in kidney disease. (a, b) The volcano maps were used to visualize the differential expressed genes between CDK vs. Normal and ESRD vs. CDK samples. The adjusted *p* value ≤ 0.05 and ∣logFC | ≥1 were considered as thresholds. Red dots represent upregulated gene while green dots represent downregulated genes. (c, d) The clustering analysis results were visualized in a heat map to identify the top 10 differential expressed genes between differential groups (CDK vs. Normal and ESRD vs. CDK).

There were 19310 genes in the GSE97709 dataset. Under the same threshold of adj.*p*.value ≤ 0.05 and ∣logFC | ≥1, we, respectively, screened 76 DEGs from CDK vs. normal samples and 10,114 DEGs from ESRD vs. CDK samples (Figures 1(a) and 1(b)). Among these genes, there were 56 upregulated and 20 downregulated genes in CDK vs. normal groups, while 4201 upregulated and 5913 downregulated genes in the ESRD vs. CDK groups. Our results indicated that genomic changes were prevalent in peripheral blood cells of ESRD patients compared with CDK samples. Furthermore, there were 51 DEGs that were significantly differentially expressed both in the CDK and ESRD groups. The top ten DEGs that were shown in the heat map represented that our cluster analysis results can significantly visualize the DEGs between the CDK vs. normal group, as well as ESRD vs. CDK group (Figures 1(c) and 1(d)).

### 3.2. GO Enrichment Analysis

GO enrichment analysis was performed for these DEGs. Firstly, there were no significantly enriched GO terms for DEGs in the CDK vs. Normal groups. Our results preliminarily indicated that there were fewer changes in gene expression patterns of CDK status. As for the numerous genes screened from the ESRD vs. CDK groups, we set the ∣logFC | ≥5 as the threshold and finally selected 1095 genes to identify related GO terms.

**Figure 2:**
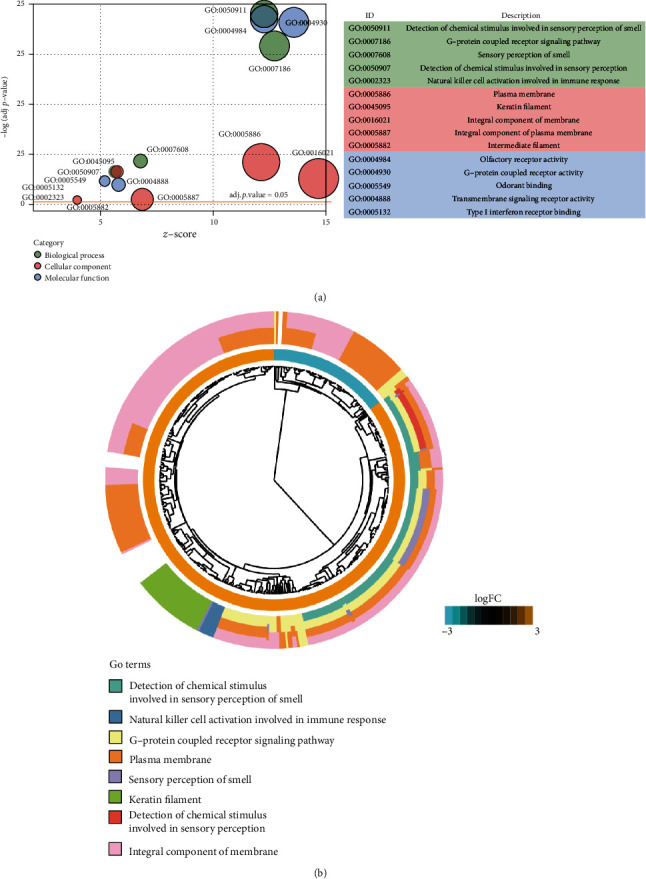
GO enrichment analysis results. (a) The top five components for biological processes, cell components, and molecular functions were visualized. (b) Selection of top 8 GO terms and related differential expressed genes for construction of highly clustering phylogenetic tree.

These DEGs were mainly enriched in 7 biological processes (BP), 6 cell components (CC), and 6 molecular functions (MF). The terms were involved in sensory perception, immune system function, and several signaling pathways, including detection of chemical stimulus involved in sensory perception of perception (GO:0050911, *p* value = 1.26*E*-99), sensory perception of smell (GO:0007608, *p* value = 1.68E-25), detection of chemical stimulus involved in sensory perception (GO:0050907, *p* value = 5.58*E*-20), natural killer cell activation involved in immune response (GO:0002323, *p* value = 8.54*E*-05), the G protein-coupled receptor protein signaling pathway (GO:0007186, *p* value = 2.93*E*-83), and adenylate cyclase-activating serotonin receptor signaling pathway (GO:0007192, *p* value = 1.18*E*-04). The top five terms were shown in Figure 2.

### 3.3. Gene Set Enrichment Analysis

**Figure 3:**
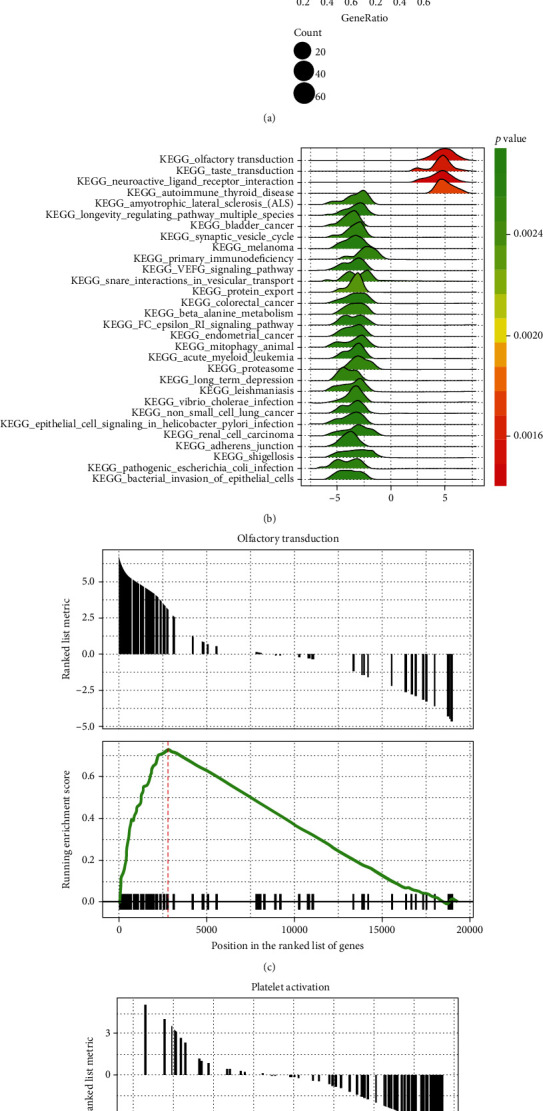
Gene set enrichment analysis results to identify crucial signaling pathways related to chronic kidney disease. (a, b) The dotplot and joyplot were constructed to visualize the up- and downregulated pathways associated to chronic kidney disease. (c, d) Representative enrichment map was visualized for pathway categories in chronic kidney disease, including upregulated pathway olfactory transduction and downregulated pathway platelet activation.

According to the GSEA method, we identified a total of 141 signaling pathways associated with kidney disease. The categories with significant differential expressed values were visualized in Figure 3. Upregulated pathways included olfactory transduction, taste transduction, neuroactive ligand-receptor interaction, and autoimmune thyroid diseases. The normalized enrichment score (NES) is the primary statistic parameter for examining gene set enrichment results. Our results showed that olfactory transduction exhibited the highest NES value (Figure 3(c)). Olfactory receptors expressed in the olfactory epithelium act a major role in olfactory transduction. Stimulation of the olfactory receptor is involved in the promotion of invasion and metastasis in cancer cells [15]. Furthermore, the mutation of proteins (NPHP6; BBS1, and BBS4) in olfactory epithelium can result in anosmia as well as renal cystic disease [16]. In addition, several significantly downregulated pathways were identified, including bacterial invasion of epithelial cells, renal cell carcinoma, bladder cancer, and phagocytic function. Among these downregulated pathways, platelet activation represented higher NES values (Figure 3(d)). Previous studies have shown that abnormal platelet activity is closely associated with CKD and plays a major role in renal failure [17–19].

### 3.4. Gene Module Mining and Weighted Gene Coexpression Network Analysis

By combining the clinical information of patients, we utilized the WGCNA methods to identify critical DEGs from ESRD vs. CDK samples. Finally, a total of 821 genes were obtained, and these genes were clustering in three modules, namely, ME1 (314 genes), ME2 (85 genes), and ME3 (83 genes). As for the genes coexpressed in no one module, we assigned these cohorts as ME0 module.

**Figure 4:**
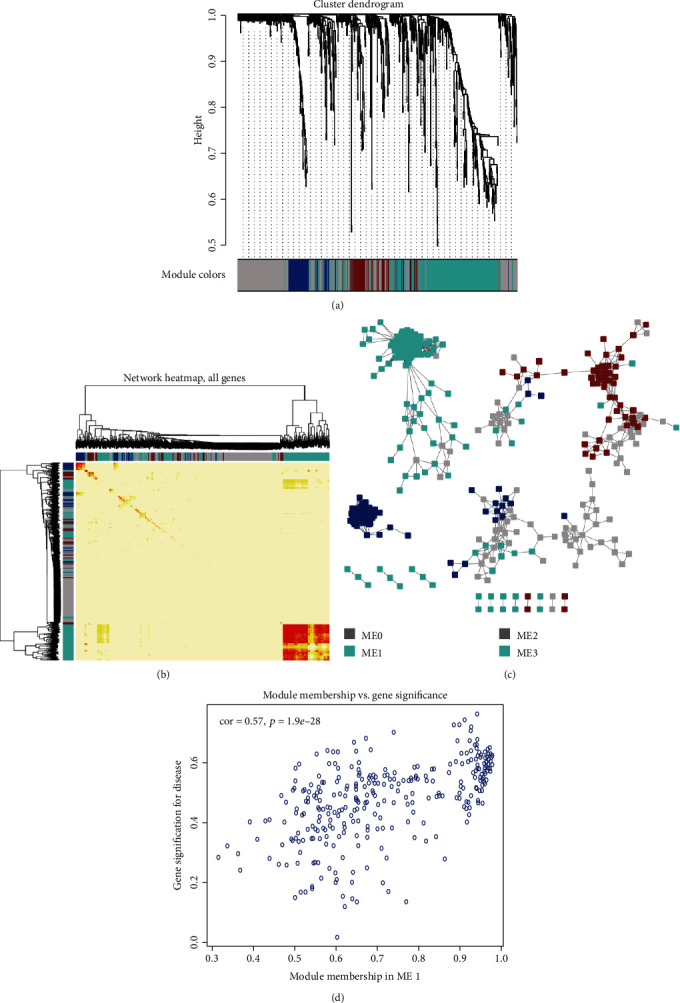
WGCNA of ESRD-related genes. (a). Hierarchical clustering dendrogram of genes. Cluster dendrogram was constructed according to gene expressed values in each sample; (b) Hierarchical clustering heat map. Gene clusters in different colors represent for correlation-ship of genes expressed values. A deep color indicated a higher correlation-ship among two genes. (c) Gene coexpression network and modules related to ESRD. In the WGCNA network, the edge weight of 0.05 is considered as a threshold value for screening modules, and points with different colors represented that the genes belong to different modules. (d) The correlation of module-membership and gene significance. The *x*-axis represents the module-membership of genes in ME1 (the values range from 0 to 1). The *y*-axis represents the gene significance for disease (the values range from 0 to 1). Regression analysis was performed on scatter plots to calculate the Pearson correlation coefficient and corresponding *p* value.

The gene connections in each module were evaluated by calculating the correlation coefficient (Table 1). It was obvious that the ME1 had the highest module significance (correlationcoefficient = −0.63439902 and adj.*p*.value = 3.86*e* − 06) and contained the largest number of DEGs (314 genes) among these modules. So, further analyses were focused on the DEGs in ME1. The results showed that numerous genes with high-connection coefficient values were identified as hub genes in the ME1 module (Figure 4(c)). The top ten genes with higher values included *TMBIM6*, *REXO1L2P*, *MAPRE1*, *LGALSL*, *DAPP1*, *CMPK1*, *BRK1*, *YWHAQ*, *TBL1XR1*, and *PDE5A*. Several genes have been shown to be involved in the pathogenesis of nephropathy, such as *TMBIM6*, *BRK1*, *YWHAQ*, and *PDE5A*; while other genes were fewer reported (*REXO1L2P*, *MAPRE1*, *LGALSL*, *DAPP1*, *CMPK1*, and *TBL1XR1*).

We also identified three lncRNAs (WWC2-AS2, SH3RF3-AS1, and DGCR9) as hub genes in the ME1 module, and coexpressed mRNA numbers were 6, 84, and 6, respectively. GO enrichment analysis was performed for these coexpressed mRNAs of SH3RF3-AS1. The biological terms were mainly associated with extracellular exosome (adj.*p*.value = 7.4*E* − 8) and focal adhesion (adj.*p*.value = 1.2*E* − 3).

### 3.5. Machine Learning Algorithm-Based Prediction Model of ESRD

**Figure 5:**
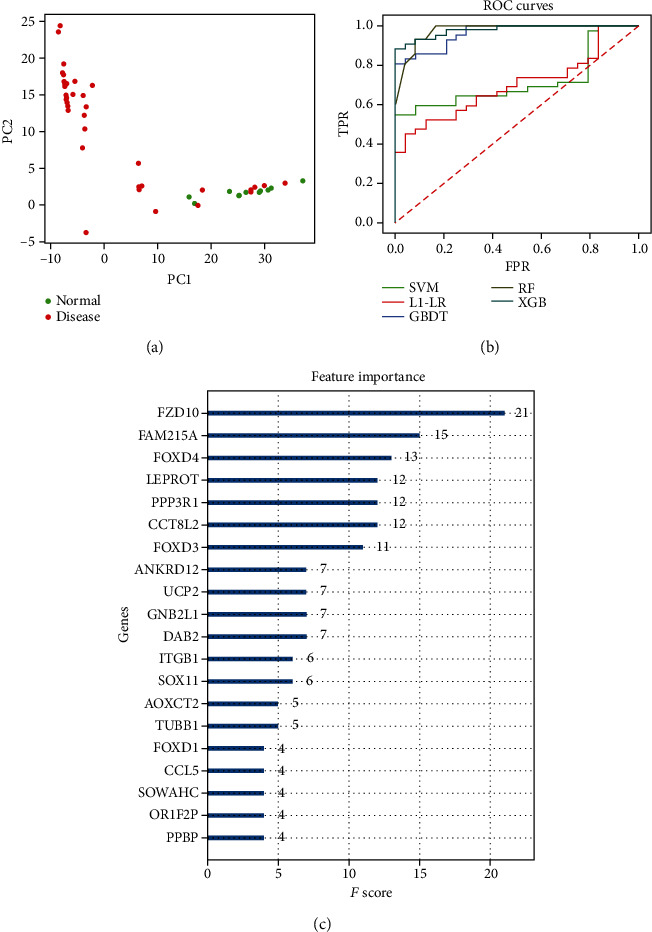
The model simulation. (a) Principal Component Analysis (PCA) results. PC1 and PC2, respectively, represent the two principal components with the largest variance value in PCA results. The points with different colors in the figures represent the Disease and Normal samples, respectively. (b) ROC curves analysis for the comparison of five training models. FPR represents False Positive Rate (FP/(FP + TN)), and TPR refers to True Positive Rate (TP/(TP + FN)). Various colors represented different training models. A larger area under ROC curves indicated a better prediction feature of models. (c) The top 20 features were evaluated by the XGB model. *F* score indicates the feature importance. A larger *F* score refers to the more importance of the gene in the XGB model.

As for the 314 genes in the ME1 module, the expressed values were extracted from two datasets (GSE97709 and GSE37171). The corresponding sample was integrated and finally obtained a total of 111 diseases (CDK and ESRD) and 52 normal samples. Principal Component Analysis (PCA) was performed according to the gene expressed values in disease samples. The results showed that gene expression values could be used as characteristics to distinguish disease samples from normal samples (Figure 5(a)).

Several training models were utilized for cross-validation of the dataset. The optimal parameters of different models were calculated (Table 2), and the results showed that three training model (GBDT, RF, and XGB) exhibited better precision and AUC values than other training models. XGB model was identified as the optimal disease prediction model in this study, in which the AUC area is 0.978, and the accuracy rate was 90%.

By calculating the *F* score in the XGBoost model [14, 20], we screened cohorts of genes associated with kidney disease progression and the top 20 genes were visualized (Figure 5(c)), such as *FZD10*, *FOXD4*, and *FAM215A*. *FZD10* represented the highest score (*F* score = 20), indicating that it played a major role in disease development.

## 4. Discussion

In this present study, we analyzed the mRNA expression profiles associated with peripheral blood samples from subjects with CDK, ESRD, and healthy control. Large cohorts of DEGs were identified from ESRD vs. CDK samples. Functional enrichment results for these DEGs revealed that olfactory transduction is markedly upregulated while the platelet activation pathway is significantly reduced in disease progression. We focused on the gene modules mining. By using the WGCNA method, several crucial genes and three lncRNAs were related to progressive CDK. Based on the XGBoost prediction model, *FZD10*, *FOXD4*, *PPP3R1*, and *UCP2* were identified as progressive-associated molecular in CDK patients.

Multiple signaling pathways were abnormally expressed in CDK progression. According to the GSEA method, platelet activation pathways were significantly downregulated while olfactory transduction was upregulated. Patients with ESRD could develop hemostatic disorders due to bleeding diatheses, and platelet dysfunction was a major factor responsible for hemorrhagic tendencies in advanced kidney disease patients [21]. Our study revealed that abnormal platelet function plays a major role in ESRD development, which was consistent with previous contents. Olfactory function has been found severely impaired in chronic renal failure patients, and the ability to discriminate and identify odors was loss in general population [22]. Olfactory deficit was involved in food aversion, anorexia, and malnutrition; and these symptoms resulted in malnutrition, which was a major factor of mortality in patients with ESRD [23, 24]. However, the potential mechanism of olfactory function loss was still unclear in patients with kidney disease. A randomized controlled trial revealed that patients with ESRD represented a higher olfactory threshold than control groups, and intranasal theophylline therapy might result in olfactory improvement [25]. Koseoglu et al. reported that nondiabetic chronic renal failure also affects olfactory functions negatively, and dialysis can improve olfactory function [26]. Olfactory receptors (ORs) are chemosensors responsible for an individual's smell function. A recent study suggested that ORs are not only expressed in the nose but also found in various other tissues including kidney tissue; one of the renal olfactory receptor (Olfr1393) might contribute to the development of type 2 diabetes according to regulate Na + -glucose cotransportation in proximal tubule [27]. Our results found that abnormal olfactory transduction pathways were identified in ESRD patient's samples, which indicated its potential regulated role in CDK progression.

By construction of the XGBoost model, we screened genes associated with progressive kidney disease, such as *FZD10*, *FOXD3/FOXD4*, and *FAM215A*. Of these genes, *FZD10* might be hub genes for the highest feature importance score. *FZD10*, also known as CD350, belongs to the frizzled gene family. Most frizzled receptors are involved in the activation of the beta-catenin pathway, and the dysregulated activation of Wnt/*β*-catenin is found in various experimental CKD models and human nephropathies [28]. Upregulated FZD10 has been reported in multiple cancer types including colon cancer, gastric cancer, and breast cancer [29], whereas the precise roles of FZD10 in CDK were fewer reported. FZD10 can interact with HIG2 protein to enhance the activation of WNT/*β*-catenin signaling, which was involved in the growth of renal cell carcinoma [30]. Inhibited FZD10 expression via leflunomide treatment or RNAi targeting also caused suppression of renal cancer cell growth [31]. Based on these findings, we promoted that FZD10 might regulate CKD progression through the *β*-catenin pathway. Furthermore, FOXD3/FOXD4 has been broadly reported as forkhead transcription factors to regulate the generation and differentiation of neural crest cells [32]. A recent study showed that FOXD4 was identified as a novel biomarker for the diagnosis and treatment of patients with CRC [33]. Serum FOXD3 expression was downregulated and associated with the diagnosis of patients with NSCLC [34]. However, whether FOXD3/FOXD4 related to kidney disease remains unclear and needs further investigation.

As for the other genes related to CKD, the function of lnRNA-*FAM215A* has been fewer investigated, and a meta-analysis report showed that the high expression of *FAM215A* was associated with longer overall survival in ovarian cancer [35]. Inhabited *FAM215A* expression results in the increasing death of melanoma cells [36]. UCP2 is an anion transporter that regulated intracellular oxidative stress. Downregulation of UCP2 has been related to several pathological sequelae in animal models, particularly affected vasculature and kidney [37]. Association analysis showed that the polymorphism of UCP2 (−866G → A) was significantly (*p* < 0.05) associated with CKD in Japanese individuals [38]. Additionally, there were still some limitations in our studies. Firstly, putative critical genes were identified according to established algorithms of the predictive model, but none of these genes has been validated experimentally in CKD. Moreover, the samples of CKD and ESRD were fewer, and more specimens should be contained as well as corresponding clinical information.

## 5. Conclusion

In summary, the results of microarray dataset analysis in our study indicate that genomic expression changes were in correlation with progressive CKD. According to prediction model construction, *FZD10*, *FOXD3/FOXD4*, *PPP3R1*, and *UCP2* were identified as progressive-associated molecular in CDK patients. Our study investigated the mechanism of genomic expression changes in CKD and promoted a new insight into the search for biomarkers with prognostic value in CDK progression.

## Figures and Tables

**Table 1:** The correlation analysis for disease and gene modules (ME1, ME2, and ME3).

	Correlation	adj.*p*.value (FDR)
ME1 vs. disease	-0.63439902	3.86*e*-06
ME2 vs. disease	0.44097924	1.71*e*-03
ME3 vs. disease	0.50514613	5.08*e*-04

Correlation: the correlation coefficient between modules and disease. FDR: false discovery rate or adjusted *p* value.

**Table 2:** The optimal parameters of different models were calculated for the validation of the test dataset.

Models	Primary parameters	Accuracy rate	AUC
SVM	Kernel = ^“^rbf^”^; *C* = 1.0; degree = 3	0.682	0.717
L1-LogisticRegression	*C* = 1.0; penalty = ^‘^l1′; max_iter = 100	0.606	0.715
GBDT	n_estimators = 100; learning_rate = 0.1; max_depth = 3; subsample = 0.8; min_samples_split = 2	0.864	0.963
RandomForest	n_estimators = 100; min_samples_leaf = 1; min_samples_split = 2	0.924	0.963
XGBoost	max_depth = 3; min_child_weight = 1; gamma = 0; learning_rate = 0.1; n_estimators = 100	0.894	0.978

## Data Availability

The microarray data used to support the findings of this study have been deposited in the GEO repository (GSE97709 and GSE37171).

## Abbreviations

CKD: Chronic kidney disease
ESRD: End-stage renal disease
DN: Diabetic nephropathy
GO: Gene ontology
CC: Cell components
MF: Molecular functions
NES: Normalized enrichment score
PCA: Principal component analysis.
